# De-embedding method for a sensing area characterization of planar microstrip sensors without evaluating error networks

**DOI:** 10.1038/s41598-024-60640-3

**Published:** 2024-05-02

**Authors:** Ugur C. Hasar, Hamdullah Ozturk, Huseyin Korkmaz, Vahid Nayyeri, Omar M. Ramahi

**Affiliations:** 1 Department of Electrical and Electronics Engineering, Gaziantep University, Gaziantep, 27310 Turkey; 2 Department of Electrical and Electronics Engineering, Gaziantep Islam Science and Technology University, Gaziantep, 27010 Turkey; 3 School of Advanced Technologies, Iran University of Science and Technology, Tehran, 1684613114 Iran; 4 Department of Electrical and Computer Engineering, University of Waterloo, Waterloo, ON N2L 3G1 Canada

**Keywords:** Planar sensors, Sensing area, S-parameters, Electrical and electronic engineering, Techniques and instrumentation

## Abstract

A de-embedding method for determining all scattering (S-) parameters (e.g., characterization) of a sensing area of planar microstrip sensors (two-port network or line) is proposed using measurements of S-parameters with no calibration. The method requires only (partially known) non-reflecting line and reflecting line standards to accomplish such a characterization. It utilizes uncalibrated S-parameter measurements of a reflecting line, direct and reversed configurations of a non-reflecting line, and direct and reversed configurations of the sensing area. As different from previous similar studies, it performs such a characterization without any sign ambiguity. The method is first validated by extracting the S-parameters of a bianisotropic metamaterial slab, as for a two-port network (line), constructed by split-ring-resonators (SRRs) from waveguide measurements. Then, it is applied for determining the S-parameters of a sensing area of a microstrip sensor involving double SRRs next to a microstrip line. The root-mean-square-error (RMSE) analysis was utilized to analyze the accuracy of our method in comparison with other techniques in the literature. It has been observed from such an analysis that our proposed de-embedding technique has the lowest RMSE values for the extracted S-parameters of the sensing area of the designed sensor in comparison with those of the compared other de-embedding techniques in the literature, and have similar RMSE values in reference to those of the thru-reflect-line calibration technique. For example, while RMSE values of real and imaginary parts of the forward reflection S-parameter of this sensing area are, respectively, around 0.0271 and 0.0279 for our de-embedding method, those of one of the compared de-embedding techniques approach as high as 0.0318 and 0.0324.

Microwave sensors are used in various fields including navigation systems^1^, bioengineering^2^, food science^3^, and civil engineering^4^. In comparison with optical and mechanical sensors, microwave sensors have unique advantages such as relatively higher sensitivity, more resistance to environmental changes (pollution, dust, and dirt), and comparatively lower cost^5^. For a broadband material characterization, various microwave sensors based on reflection-transmission measurements such as conventional waveguide/coaxial line measurements, free-space methods, open-ended waveguide or coaxial line measurements, and planar structure measurements can be utilized. For example, conventional microwave waveguide methods are highly accurate. However, they are bulky require accurate and elaborate sample machining to eliminate gap effect between waveguide walls and sample lateral surfaces^6,7^. To alleviate sample preparation process, free-space methods can be used^8–10^. Nonetheless, these methods necessitate a sample transverse area greater than the foot print of the antenna at the examined frequency to eliminate diffraction effects at the sample corners or edges. Besides, open-ended waveguide or coaxial measurements can be conveniently implemented especially for liquid samples or samples with planar surfaces^11,12^. These measurements necessitate, in general, close contact with the sample under test. Additionally, theoretical analysis essentially assumes that the sample is semi-infinite and extends to infinity at the probe opening^12^. On the other hand, microwave sensors based on planar topologies take advantage of being low profile and relatively inexpensive, allowing ease of fabrication, and providing a simple means of sensing or characterization by measuring the effect of the sample near the sensing area^13–20^.

Measurement setups used in sensor applications in general require some sort of calibration before starting to measurements. Depending on criteria of applicability, feasibility, bandwidth, and accuracy, a suitable calibration procedure should be applied to eliminate systematic errors in the measurement system. While short-open-load-thru (SOLT) and short-open-load-reciprocal (SOLR) calibration techniques are convenient for coaxial line configurations, thru-reflect-line (TRL), multiline TRL, line-reflect-line (LRL), thru-reflect-match (TRM), line-reflect-match (LRM), thru-match-reflect-reflect (TMRR), and line-reflect-reflect-match (LRRM) and sliding short calibration techniques are feasible for probe wafer and waveguide configurations^21–26^. The common goal of these techniques is to first determine error networks between the vector network analyzer (VNA) and the device under test using some calibration standards with well-known characteristics. These techniques use at least three (partially or fully known) standards for device characterization.

In addition to calibration techniques some of which are presented above, de-embedding methods based on relative measurements could also be applied for a direct transmission line or sample characterization^27–45^ or a direct two-port device (or network) characterization^46–49^. These methods, contrary to calibration techniques, do not require an explicit solution of error networks or coefficients to characterize a transmission line, sample, or full two-port device or network (or line)^46^. Because the de-embedding methods in the studies^27–45^ are limited to a transmission line or sample characterization, from this point on we will mainly focus on the de-embedding methods in the studies^46–49^ which can be applied for a direct two-port device (or network) characterization (see Table 1). The de-embedding method proposed in the study^46^ was based on uncalibrated scattering (S-) parameter measurements of a thru, a non-reflecting line, and the two-port network (a coplanar waveguide discontinuity). However, it considers that the network has reflection-symmetric property. To generalize this methodology for a reflection-asymmetric two-port network, we applied a methodology relying on uncalibrated measurements of a thru, a non-reflecting line, and the two configurations of the device (direct and reversed configurations)^47^. Although it is possible to extract forward and backward S-parameters $S_{11}$ and $S_{22}$ of a device (in addition to its forward and backward transmission S-parameters $S_{21}$ and $S_{12}$), there are two sign ambiguities in the expressions of $S_{11}$ and $S_{22}$ (four solutions for each of $S_{11}$ and $S_{22}$). This necessitates a prior knowledge of $S_{11}$ and $S_{22}$ to resolve this sign ambiguity. To eliminate this drawback, we also proposed two de-embedding methods^48,49^. While the first one^48^ uses uncalibrated S-parameters of a thru, a non-reflecting line, the device, and a reflecting reference material next to the device, the second one utilizes uncalibrated S-parameters of a thru, a non-reflecting line, the direct and reversed configurations of the device. They either fail to remove the sign ambiguity in $S_{11}$ and $S_{22}$ measurements at some discrete frequencies or reduce the sign ambiguity problem to two possible solutions of $S_{11}$ or $S_{22}$.

**Figure 1:**
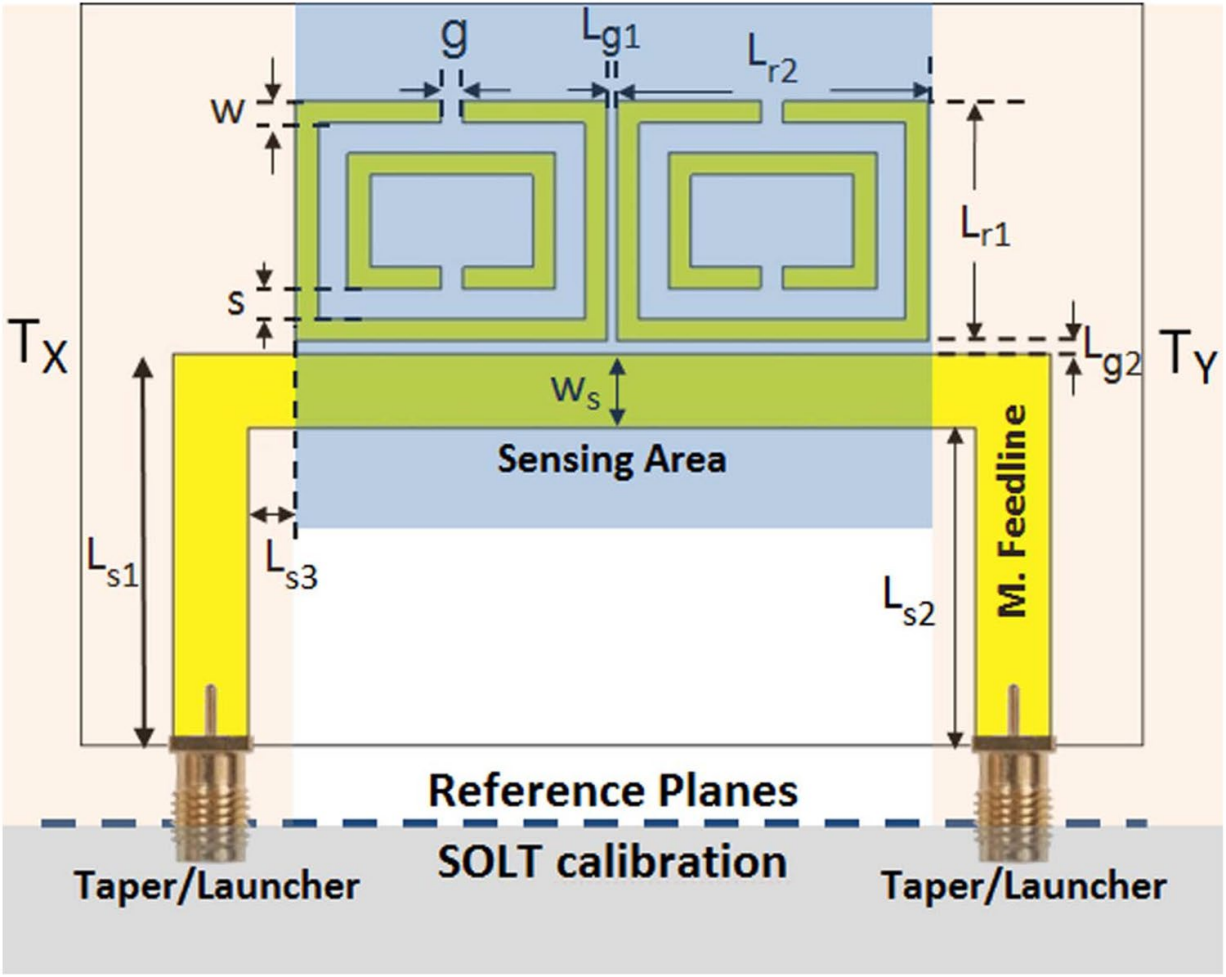
A planar microstrip sensor with a sensing area (double ring resonators) with feedlines and SMA tapers or launchers. Here, $T_X$ and $T_Y$ account for the effects of microstrip lines, SMA tapers or lauchers, coaxial lines with SMA connectors, and VNA systematic errors.

Many of the recent microwave sensor applications require planar resonators (or sensors)^13–20^ to detect a variety of sample properties. SMA connectors are generally used for carrying electromagnetic signals from a VNA through coaxial lines to the planar structure. Calibration techniques such as SOLT or SOLR can be applied to eliminate systematic errors to the end of the SMA connectors or to the SMA taper or launcher^50^, as shown in Fig. 1. Nonetheless, it fails to fully remove the effects of SMA taper/launcher and microstrip feed lines for an accurate full two-port sensing area (device or network (or line)) characterization. In a recent study^13^, the effects of SMA tapers/launchers were de-embedded from planar microwave sensor measurements by applying a simple procedure based on the T-matrix approach. However, this procedure necessitates some simplifications in the theoretical analysis. First, it assumed that the reflection coefficient was much smaller than the transmission coefficient at the SMA connector. Second, it assumed that the SMA tapers or launchers welded to the stripline were identical. Although both of these assumptions might be in general valid for a typical SMA taper or launcher section, for a more accurate measurements, a theoretical model taking into account the case that these two assumptions may not be satisfied should be addressed. Besides, TRL, LRL, TRM, LRM, TMRR, and LRRM techniques with standards implemented directly at the microstrip section could be effectively applied for removing the effects of launchers/tapers and even microstrip feedlines next to the sensing area (see Fig. 1). However, they require at least three different calibration standards to evaluate error networks prior to a full two-port characterization of the sensing area (see Table 1). Our concern in this study is to perform a full two-port characterization of a sensing area, as for the two-port device or network shown in Fig. 1, of planar microstrip sensors (or in general planar microwave sensors) by a de-embedding technique using uncalibrated S-parameters. Such a characterization is a necessity for a more accurate sample property analysis. To meet such a requirement, in this study, we propose a deembeeding method to uniquely extract (without any sign ambiguity) all S-parameters of the sensing area of microstrip sensors (see Fig. 1) from uncalibrated (raw) S-parameter measurements of (partially known) two different standards without the need for evaluating error networks a priori. As standards, a reflecting line (with partial reflection) and direct and reversed configurations of a non-reflecting line, whose propagation constant can be determined, are utilized.

**Table 1:** Comparison of the proposed method (‘PM’) with other calibration and de-embedding techniques in the literature.

	Calibration Technique		De-embedding Technique		
Parameter	SOLT^21^	TRL^22^ (or LRL)	^46^	^47–49^	PM
Error network Analysis	Yes, needed to evaluate		No need to evaluate		
Number of Standards	4	3	2	2 or 3	2
Full Two-Port Characterization	Yes		No	Yes	Yes
Sign Ambiguity	No		Yes	Sometimes	No
Realization of Standards	Partly difficult	Simpler	Simpler		
New Design Requirement	No		Yes		

## The analysis of the method

**Figure 2:**
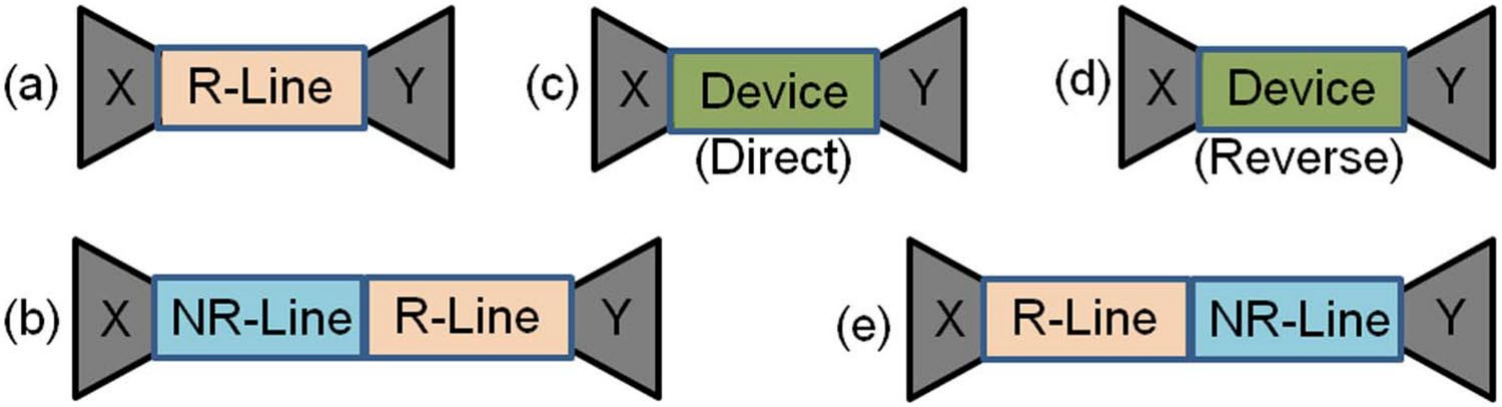
Measurement configurations: (**a**) A reflecting line (R-Line) between error networks *X* and *Y*, (**b**) a non-reflecting line (NR-Line) next to the R-Line between *X* and *Y*, (**c**) and (**d**) direct and reversed connections of a device between *X* and *Y*, and (**e**) the reversed configuration in (**b**).

Five different measurement configurations composing of two different standards and one device in the implementation of our method are schematically depicted in Fig. 2. Figure 2a corresponds to the configuration where a (reciprocal) reflecting line (R-Line) is connected between two unknown error networks *X* and *Y*, which are complex functions of VNA source and load mismatches, impedance change at the connections, SMA connectors and tapers or launchers, feed lines, etc. Figure 2b illustrates the configuration where a non-reflecting line (NR-Line) is positioned next to the R-Line between *X* and *Y*. Figure 2c,d present the direct and reversed configurations of the device between *X* and *Y*. Finally, Fig. 2e demonstrates the reversed configuration in Fig. 2b.

### Wave-cascaded matrix representation

The well-known wave-cascaded matrix (WCM) representation based on 8-term error model (directivity, source match, reflecting tracking, transmission) could be used to examine the theoretical analysis^22^. For the configurations in Fig. 2a–e, one obtains

1 $$\begin{aligned} M_a= & {} T_X T_{\text {RL}} T_Y, \ \ M_b = T_X T_{\text {NRL}} T_{\text {RL}} T_Y, \ \ M_c = T_X T_D T_Y, \end{aligned}$$

2 $$\begin{aligned} M_d= & {} T_X T_D^{\text {inv}} T_Y, \ \ M_e = T_X T_{\text {RL}} T_{\text {NRL}} T_Y, \end{aligned}$$

where $M_a$, $M_b$, $M_c$, $M_d$, and $M_e$ are the WCMs corresponding to the configurations in Fig. 2a–e, respectively; $T_X$ and $T_Y$ are the WCMs of the error networks *X* and *Y*; $T_{\text {RL}}$ and $T_{\text {NRL}}$ are the WCMs of the reflecting- and non-reflecting lines; and $T_D$ and $T_D^{\text {inv}}$ are the WCMs of the direct and reversed configurations of the device. $M_a$, $M_b$, $M_c$, $M_d$, and $M_e$ are related to measured S-parameters:

3 $$\begin{aligned} M_k = \frac{1}{S_{21k}} \begin{bmatrix} S_{21k} S_{12k} - S_{11k} S_{22k} &{} S_{11k} \\ -S_{22k} &{} 1 \end{bmatrix}, \end{aligned}$$

where *k* is *a*, *b*, *c*, *d*, or *e*. Using (3), it is possible to express $T_{\text {RL}}$, $T_{\text {NRL}}$, $T_D$, and $T_D^{\text {inv}}$ as

4 $$\begin{aligned} T_{\text {RL}}&= \frac{1}{ (1 - \Gamma ^2) P} \begin{bmatrix} P^2 - \Gamma ^2 &{} \Gamma (1 - P^2) \\ -\Gamma (1 - P^2) &{} 1 - \Gamma ^2 P^2 \end{bmatrix} \nonumber \\&= \begin{bmatrix} \Omega _1 &{} \Omega _2 \\ -\Omega _2 &{} \Omega _3 \end{bmatrix}, \ \ \ \ T_{\text {NRL}} = \begin{bmatrix} P_0 &{} 0 \\ 0 &{} 1/P_0 \end{bmatrix}, \end{aligned}$$

5 $$\begin{aligned} \Gamma&= (z_{\text {eff}} - 1)/(z_{\text {eff}} + 1), \ \ \ P = e^{- \gamma _{\text {eff}} L_r}, \ \ P_0 = e^{- \gamma _{\text {eff,0}} L_{nr}}, \end{aligned}$$

6 $$\begin{aligned} \gamma _{\text {eff}}&= i k_0 \sqrt{\varepsilon _{\text {eff}}}, \ \ \ \gamma _{\text {eff,0}} = i k_0 \sqrt{\varepsilon _{\text {eff,0}}}, \end{aligned}$$

7 $$\begin{aligned} T_D&= \frac{1}{S_{21}^D} \begin{bmatrix} S_{21}^D S_{12}^D - S_{11}^D S_{22}^D &{} S_{11}^D \\ -S_{22}^D &{} 1 \end{bmatrix} = \frac{1}{S_{21}^D} \begin{bmatrix} -\Delta _D &{} S_{11}^D \\ -S_{22}^D &{} 1 \end{bmatrix}, \end{aligned}$$

8 $$\begin{aligned} T_D^{\text {inv}}&= \frac{1}{S_{12}^D} \begin{bmatrix} -\Delta _D &{} S_{22}^D \\ -S_{11}^D &{} 1 \end{bmatrix}, \ \ \Delta _D = S_{11}^D S_{22}^D - S_{21}^D S_{12}^D. \end{aligned}$$

Here, *P* and $\Gamma$ are the propagation factor of and the first reflection coefficient at the R-Line; $P_0$ is the propagation factor of the NR-Line; $z_{\text {eff}}$, $\gamma _{\text {eff}}$, $\varepsilon _{\text {eff}}$, and $L_{r}$ are the effective normalized impedance, effective propagation constant, effective permittivity, and length of the R-Line while $\gamma _{\text {eff,0}}$, $\varepsilon _{\text {eff,0}}$, and $L_{nr}$ are the effective propagation constant, effective permittivity, and length of the NR-Line; and $S_{11}^D$, $S_{21}^D$, $S_{12}^D$, and $S_{22}^D$ are the S-parameters of the device.

### Elimination of the effects of error matrices X and Y

Using WCMs in (1) and (2), it is possible to eliminate $T_Y$^28,30^:

9 $$\begin{aligned} M_b M_a^{-1}= & {} T_X T_{\text {NRL}} T_X^{-1}, \end{aligned}$$

10 $$\begin{aligned} M_e M_b^{-1}= & {} T_X T_{\text {RL}} T_{\text {NRL}} T_{\text {RL}}^{-1} T_{\text {NRL}}^{-1} T_X^{-1}, \end{aligned}$$

11 $$\begin{aligned} M_c M_b^{-1}= & {} T_X T_D T_{\text {RL}}^{-1} T_{\text {NRL}}^{-1} T_X^{-1}, \end{aligned}$$

12 $$\begin{aligned} M_d M_b^{-1}= & {} T_X T_D^{\text {inv}} T_{\text {RL}}^{-1} T_{\text {NRL}}^{-1} T_X^{-1}, \end{aligned}$$

13 $$\begin{aligned} M_c M_e^{-1}= & {} T_X T_D T_{\text {NRL}}^{-1} T_{\text {RL}}^{-1} T_X^{-1}, \end{aligned}$$

14 $$\begin{aligned} M_c M_a^{-1}= & {} T_X T_D T_{\text {RL}}^{-1} T_X^{-1}, \end{aligned}$$

15 $$\begin{aligned} M_d M_a^{-1}= & {} T_X T_D^{\text {inv}} T_{\text {RL}}^{-1} T_X^{-1}, \end{aligned}$$

where ‘$\star ^{-1}$’ denotes the inverse of the square matrix ‘$\star$’.

Using the trace operation of a square matrix, which corresponds to the sum of its eigenvalues, one can eliminate the effect of $T_X$ and determine^28,30^

16 $$\begin{aligned} \Lambda _0= & {} \text {Tr}( M_b M_a^{-1} ) = \text {Tr}( T_{\text {NRL}} ), \end{aligned}$$

17 $$\begin{aligned} \Lambda _1= & {} \text {Tr}( M_e M_b^{-1} ) = \text {Tr}( T_{\text {RL}} T_{\text {NRL}} T_{\text {RL}}^{-1} T_{\text {NRL}}^{-1}), \end{aligned}$$

18 $$\begin{aligned} \Lambda _2= & {} \text {Tr}( M_c M_b^{-1} ) = \text {Tr}( T_D T_{\text {RL}}^{-1} T_{\text {NRL}}^{-1} ), \end{aligned}$$

19 $$\begin{aligned} \Lambda _3= & {} \text {Tr}( M_d M_b^{-1} ) = \text {Tr}( T_D^{\text {inv}} T_{\text {RL}}^{-1} T_{\text {NRL}}^{-1} ), \end{aligned}$$

20 $$\begin{aligned} \Lambda _4= & {} \text {Tr}( M_c M_e^{-1} ) = \text {Tr}( T_D T_{\text {NRL}}^{-1} T_{\text {RL}}^{-1} ), \end{aligned}$$

21 $$\begin{aligned} \Lambda _5= & {} \text {Tr}( M_c M_a^{-1} ) = \text {Tr}( T_D T_{\text {RL}}^{-1} ), \end{aligned}$$

22 $$\begin{aligned} \Lambda _6= & {} \text {Tr}( M_d M_a^{-1} ) = \text {Tr}( T_D^{\text {inv}} T_{\text {RL}}^{-1} ). \end{aligned}$$

### Obtaining information about calibration standards

We determined from (4) and (16)

23 $$\begin{aligned} P_0 = e^{ - \gamma _{\text {eff,0}} L_{nr} } = \Lambda _0/2 {\mp } \sqrt{ \left( \Lambda _0 / 2 \right) ^2 - 1 }. \end{aligned}$$

Correct sign in (23) can be specified after evaluating $\gamma _{\text {eff,0}}$ and enforcing $\Re e \{ \gamma _{\text {eff,0}} \} \ge 0$ for a passive non-reflecting line. In fact, it is possible to obtain from (4) and (17)

24 $$\begin{aligned} \Lambda _1 = 2 \Omega _1 \Omega _3 + (P_0^2 + 1/P_0^2) \Omega _2^2. \end{aligned}$$

Taking into account that the reflecting line is reciprocal (that is, $\Omega _1 \Omega _3 + \Omega _2^2 = 1$), then one can derive

25 $$\begin{aligned} \Omega _2^2 = (\Lambda _1 - 2)/( P_0^2 + 1/P_0^2 - 2 ). \end{aligned}$$

This means that considering (3) and (4), only one information about the S-parameters (e.g., $S_{11}$ or $S_{21}$) of a reflecting reciprocal line is needed in the implementation of our method.

### Determination of S-parameters of the device

Substituting (4)–(8) into (16)–(22), one can evaluate

26 $$\begin{aligned} \Lambda _2= & {} \big [ P_0 ( \Omega _1 + \Omega _2 S_{22}^D) + (-\Omega _3 \Delta _D + \Omega _2 S_{11}^D)/P_0 \big ] / S_{21}^D, \end{aligned}$$

27 $$\begin{aligned} \Lambda _3= & {} \big [ P_0 ( \Omega _1 + \Omega _2 S_{11}^D) + (-\Omega _3 \Delta _D + \Omega _2 S_{22}^D)/P_0 \big ] / S_{12}^D, \end{aligned}$$

28 $$\begin{aligned} \Lambda _4= & {} \big [ P_0 ( \Omega _1 + \Omega _2 S_{11}^D) + (-\Omega _3 \Delta _D + \Omega _2 S_{22}^D)/P_0 \big ] / S_{21}^D, \end{aligned}$$

29 $$\begin{aligned} \Lambda _5= & {} \big [ ( \Omega _1 + \Omega _2 S_{22}^D) + (-\Omega _3 \Delta _D + \Omega _2 S_{11}^D) \big ] / S_{21}^D, \end{aligned}$$

30 $$\begin{aligned} \Lambda _6= & {} \big [ ( \Omega _1 + \Omega _2 S_{11}^D) + (-\Omega _3 \Delta _D + \Omega _2 S_{22}^D) \big ] / S_{12}^D. \end{aligned}$$

From (26)–(30), one can determine

31 $$\begin{aligned} \Psi _1= & {} \frac{\Lambda _2}{\Lambda _4} = \frac{ P_0^2 ( \Omega _1 + \Omega _2 S_{22}^D) + (-\Omega _3 \Delta _D + \Omega _2 S_{11}^D) }{ P_0^2 ( \Omega _1 + \Omega _2 S_{11}^D) + (-\Omega _3 \Delta _D + \Omega _2 S_{22}^D) }, \end{aligned}$$

32 $$\begin{aligned} \Psi _2= & {} \frac{\Lambda _2}{\Lambda _5} = \frac{ P_0^2 ( \Omega _1 + \Omega _2 S_{22}^D) + (-\Omega _3 \Delta _D + \Omega _2 S_{11}^D) }{ P_0 \big [ ( \Omega _1 + \Omega _2 S_{22}^D) + (-\Omega _3 \Delta _D + \Omega _2 S_{11}^D ) \big ] }, \end{aligned}$$

33 $$\begin{aligned} \Psi _3= & {} \frac{\Lambda _3}{\Lambda _6} = \frac{ P_0^2 ( \Omega _1 + \Omega _2 S_{11}^D) + (-\Omega _3 \Delta _D + \Omega _2 S_{22}^D) }{ P_0 \big [ ( \Omega _1 + \Omega _2 S_{11}^D) + (-\Omega _3 \Delta _D + \Omega _2 S_{22}^D ) \big ] }, \end{aligned}$$

where $P_0$ can be ascertained from (23).

One can find $\Delta _D$ from (31)–(33) that

34 $$\begin{aligned} \Delta _D&= \frac{ P_0^2 \Omega _1 (1 - \Psi _1) + \Omega _2 (1 - P_0^2 \Psi _1) S_{11}^D + \Omega _2 (P_0^2 - \Psi _1) S_{22}^D }{ \Omega _3 (1 - \Psi _1) } \nonumber \\&= \frac{ \Omega _1 (P_0 - \Psi _2) + \Omega _2 (1/P_0 - \Psi _2) S_{11}^D + \Omega _2 (P_0 - \Psi _2) S_{22}^D }{ \Omega _3 (1/P_0 - \Psi _2) } \nonumber \\&= \frac{ \Omega _1 (P_0 - \Psi _3) + \Omega _2 (P_0 - \Psi _3) S_{11}^D + \Omega _2 (1/P_0 - \Psi _3) S_{22}^D }{ \Omega _3 (1/P_0 - \Psi _3) }. \end{aligned}$$

Using the equality in (34), one obtains $S_{22}^D$

35 $$\begin{aligned} S_{22}^D= & {} ( \Phi _2 S_{11}^D + \Phi _3 )/\Phi _1 = ( \Phi _5 S_{11}^D + \Phi _6 )/\Phi _4, \end{aligned}$$

36 $$\begin{aligned} \Phi _1= & {} \frac{(1 - P_0^2)}{(1 - \Psi _1)} \frac{(\Psi _1 - P_0 \Psi _2)}{(1 - P_0 \Psi _2)}, \end{aligned}$$

37 $$\begin{aligned} \Phi _2= & {} \Psi _1 \left( \frac{1 - P_0^2}{1 - \Psi _1} \right) , \ \ \Phi _3 = \frac{\Omega _1}{\Omega _2} \frac{P_0 \Psi _2 (1 - P_0^2)}{(1 - P_0 \Psi _2)}, \end{aligned}$$

38 $$\begin{aligned} \Phi _4= & {} 1 - P_0 \Psi _3, \ \ \Phi _5 = 1 - P_0 \Psi _2, \end{aligned}$$

39 $$\begin{aligned} \Phi _6= & {} \frac{\Omega _1}{\Omega _2} P_0 ( \Psi _3 - \Psi _2 ). \end{aligned}$$

Here, $\Omega _1$ and $\Omega _2$ are the quantities in functions of $\Gamma$ and *P* of the reflecting line in (4) and (5).

After, $S_{11}^D$ is uniquely found from (35)

40 $$\begin{aligned} S_{11}^D = (\Phi _1 \Phi _6 - \Phi _3 \Phi _4 )/( \Phi _2 \Phi _4 - \Phi _1 \Phi _5 ). \end{aligned}$$

Once $S_{11}^D$ is calculated from (40), $S_{22}^D$ and $\Delta _D$ can be determined in a simple manner from (34) and (35). Finally, $S_{21}^D$ and $S_{12}^D$ can be evaluated from (26)–(30). It is noted that (23) can be utilized to determine $\gamma _{\text {eff,0}}$ as a byproduct.

Finally, it should be stressed that when $P_0$ approaches unity, as other calibration methods such as the TRL calibration technique^22^, our proposed method breaks down ($\Lambda _2 = \Lambda _4 = \Lambda _5$ and $\Lambda _3 = \Lambda _6$). Therefore, it is not possible to determine meaningful $S_{11}^D$, $S_{21}^D$, $S_{12}^D$, and $S_{22}^D$ from (26)–(40). Discussion of this point is given in Section *Validation*.

## Validation

**Figure 3:**
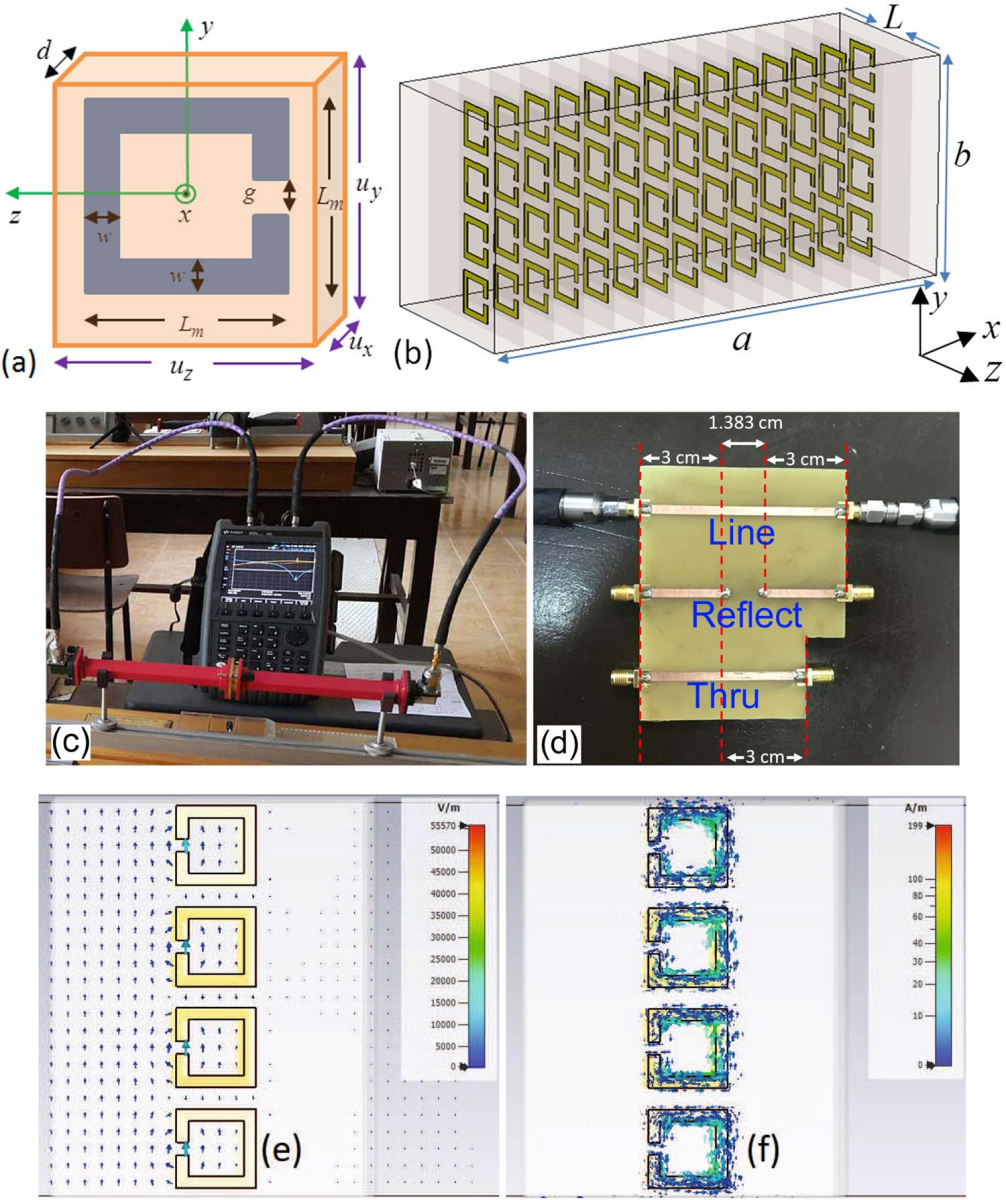
(**a**) Dimensions of the examined cell, (**b**) the MM slab formed by cascaded connection of fourteen individual units with length $L_{sub} = 8.10$ mm, each of which has four SRRs along $y-$direction (*a* and *b*, respectively, refer to broader and narrower dimensions of the waveguide cross section)^52^, (**c**) a picture of the measurement setup operating at X-band, (**d**) a picture of the designed TRL calibration kit for microstrip measurements, (**e**) electric field distribution around the SRRs (side view), and (**f**) surface current distribution on the surface of the metals of SRRs (side view) at 11.867 GHz.

### Measurement setup

The rectangular waveguide setup operated at X-band (8.2- 12.4 GHz, $a = 22.86$ mm, $b = 10.16$ mm, and $f_c = 6.557$ GHz) was constructed for validation (see Fig. 3c). The VNA used in our measurements (Keysight Technologies – N9918A) has a frequency range between 30 kHz and 26.5 GHz. Two longer phase stable coaxial lines were employed to carry signals. Besides, two coax-to-waveguide adapters were secured to two longer additional waveguide straights (approximately 200 mm) to suppress high-order modes, if present. Details about the measurement setup are available in^51^.

### Constructed bianisotropic metamaterial (MM) Slab

S-parameters of a bianisotropic metamaterial (MM) slab loaded into an X-band rectangular waveguide section, considered as for the device, were performed for the validation of our method. This slab was constructed by a unit cell with a square edge-coupled split-ring-resonator (SRR), as shown in Fig. 3a with the following geometrical parameters: $L_m = 2.00$ mm, $w = g = 0.30$ mm, $u_x = d + t_m$, $u_y = 2.54$ mm, and $u_z = L_{sub} = 8.10$ mm. Here, $t_m = 35\mu$m corresponds to the metal thickness (copper material with conductivity $\sigma = 5.8 \times 10^7$ S/m) while $d = 1.50$ mm and $L_{sub}$ denote, respectively, the substrate thickness and length (FR4 material with $\varepsilon _{r,sub} = 4.3 (1 - i 0.025)$). Each sub-unit (four SRRs positioned in the $y-$direction) was fabricated using the conventional printed circuit technology^51^. As shown in Fig. 3b, the MM slab was formed by locating fourteen sub-units in cascaded manner in the $x-$direction. This slab is identical to that in the study^52^, which is just used here for validation.

The reason for using four SRRs in the $y-$direction, with fourteen times repetition in the $x-$direction, was to ensure a homogeneous material. According to the effective medium theory^51^, repetition periodicity of the unit cell over a transverse plane should be smaller than one-tenth of the wavelength in order for the intrinsically inhomogeneous MM slab to be considered as a homogeneous MM slab. In our case, the constructed MM slab has $u_x = 1.535$ mm and $u_y = 2.54$ mm where $u_x$ and $u_y$ are the periodicities in the $x-$ and $y-$directions. Both $u_x$ and $u_y$ are considerably less than the wavelength of free-space (around 30 mm) at the middle frequency. This means that the MM slab satisfies the effective medium assumption. Two extra FR4 substrates (without any metallic design) with $10.16 \times 8.10 \times 0.55$ mm^3^ were inserted at the left and right side guide walls for eliminating air gap effect^51,52^.

While electromagnetic wave is propagating through the MM slab inside a rectangular waveguide, it interacts with the edge-coupled SRRs. This interaction interaction occurs in the following manner^53^. Electric field of the dominant TE_10_ mode in the $y-$axis (normal to the slit axis) forces charges with opposite polarities to accumulate at opposite sides (w.r.t. the $z-$axis) of both rings (electric excitation). This will in turn produce circulating currents and then create a magnetic dipole in the $x-$axis. Figure 3e illustrates electric field distribution (at the instant of maximum variation) on the plane of SRRs (electric flux lines originating from and ending with the SRRs) at the frequency of 11.867 GHz around which transmission S-parameter ($S_{21}$) has a dip^51^. Besides, magnetic field of the dominant TE_10_ mode in the $x-$direction, which is normal to the plane of SRRs, influences charges to circulate within the metal of the SRRs (magnetic excitation). This in turn will induce a non-zero net electric dipole moment in the $y-$axis. Figure 3f presents surface current distribution (at the instant of maximum variation) on the surface of the metals of SRRs (circulating current) at the same frequency (11.867 GHz). As a consequence of such coupling mechanism of electric and magnetic fields over the waveguide cross section, a non-zero magneto-electric coupling will be present^53^, resulting in a non-identical forward and backward reflection S-parameters^51^.

### Analysis

**Figure 4:**
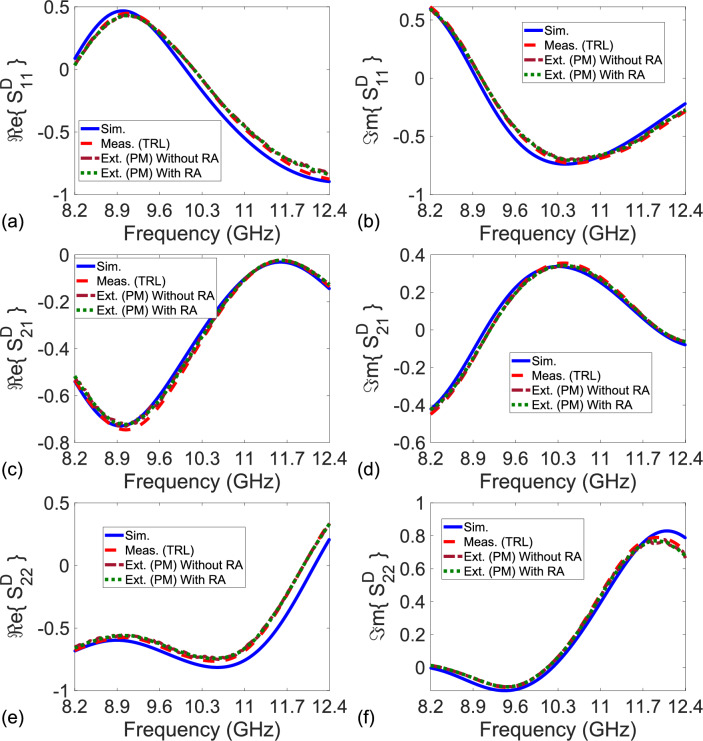
Simulated S-parameters (‘Sim.’ with solid lines), measured S-parameters after the TRL calibration technique (‘Meas. (TRL)’ with dashed lines), and extracted S-parameters by the proposed method for $L_{nr} = 9.4$ mm (‘Ext. (PM) Without RA’ by dashdot lines for the result without RA and ‘Ext. (PM) With RA’ by dotted lines for the result with RA) of the constructed bianisotropic MM slab. (**a**) Real and (**b**) imaginary parts of $S_{11}^D$, (**c**) real and (**d**) imaginary parts of $S_{21}^D$ ($\cong S_{12}^D$), and (**e**) real and (**f**) imaginary parts of $S_{22}^D$.

Figure 4a–f illustrate the simulated S-parameters (‘Sim.’ with solid lines), measured S-parameters after the TRL calibration (‘Meas. (TRL)’ with dashed lines), and extracted S-parameters by the proposed method (‘Ext. (PM)’ with dotted lines) of the constructed bianisotropic MM slab. In application of the TRL calibration technique^22^, a waveguide section with a length of 9.4 mm was utilized as for the line standard. Then, calibrated S-parameters of the constructed bianisotropic MM slab were measured. In application of our method, we implemented the measurement configurations in Fig. 2a–e using uncalibrated S-parameter measurements with (and without) the rolling average (RA) procedure applied for frequency range of approximately 42 MHz^54^ calculated from $N_{\text {int}} (f_{\text {max}} - f_{\text {min}})/N_f$ where $N_{\text {int}}$, $f_{\text {max}}$, $f_{\text {min}}$, and $N_f$ denote, respectively, the number of intervals (the number of frequency points), maximum and minimum frequencies the measurements are conducted, and the number of total intervals (frequency points). In measurements, $N_{\text {int}} = 10$ (deliberately selected partly greater than the value used in the study^54^ for better smoothed data), $f_{\text {max}} = 12.4$ GHz, $f_{\text {min}} = 8.2$ GHz, and $N_f = 1001$.

While an empty waveguide section with a length of $L_{nr} = 9.4$ mm was used as for the NR-Line, a waveguide section with a length of $L_r = 7.7$ mm with a polyethylene (PE) sample (3.85 mm) flushed at its right terminal was considered as the R-Line. In selection of the length of the NR-Line, as discussed in Section *The Analysis of the Method*, we considered the point that $P_0$ does not approach unity. In obtaining simulated S-parameters, the Computer Simulation Technology (CST) Microwave Studio was utilized^51^. $E_t = 0$ boundary conditions were applied over the transverse plane ($x = 0$, $x = a$, $y = 0$, and $y = b$ planes) to imitate hollow metallic waveguide. Waveguide ports were positioned at appropriate positions at the $z-$direction. The adaptive mesh option was set active in the solver with an accuracy of $10^{-12}$ (3^rd^ order solver).

**Figure 5:**
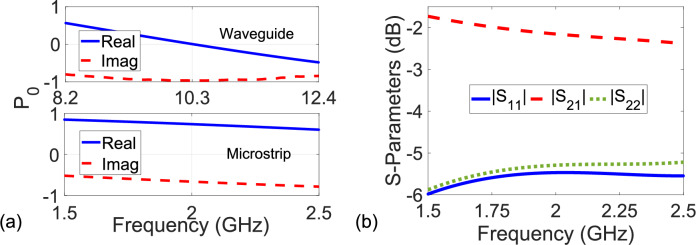
(**a**) Real and imaginary parts of measured $P_0$ for the NR-Line (an empty waveguide section) with $L_{nr} = 9.4$ mm and the NR-Line composed of a microstrip line with $L_{nr} = 9.7$ mm and (**b**) magnitudes of simulated S-parameters of the configuration in Fig. 7b.

It is noted from Fig. 4a–f that simulated, measured, and extracted S-parameters of the MM slab ($S_{21}^D \cong S_{12}^D$), which has $S_{11}^D \ne S_{22}^D$ due to bianisotropic behavior^51^, are in good agreement with each other over entire frequency band. This validates our proposed method. Relatively smaller discrepancies between the simulated and measured/extracted S-parameters are chiefly a cause of fabrication process^51^. Because our method assumes that $P_0 \ne 1.0$, it would be instructive to examine its behavior. Figure 5a demonstrates the real and imaginary parts of $P_0$ of the used NR-Line with length $L_{nr} = 9.4$ mm over frequency. It is seen from Fig. 5a that $P_0$ differs from unity over the entire frequency band.

**Figure 6:**
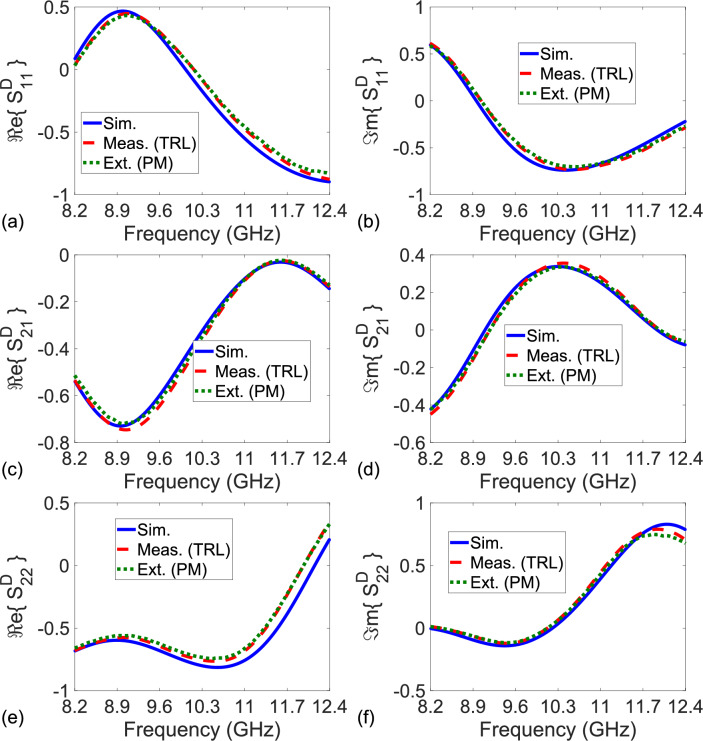
Simulated S-parameters (‘Sim.’ with solid lines), measured S-parameters after the TRL calibration technique (‘Meas. (TRL)’ with dashed lines), and extracted S-parameters using uncalibrated measurements by the proposed method for $L_{nr} = 10.16$ mm (‘Ext. (PM)’ with dotted lines) of the constructed bianisotropic MM slab. (**a**) Real and (**b**) imaginary parts of $S_{11}^D$, (**c**) real and (**d**) imaginary parts of $S_{21}^D$ ($\cong S_{12}^D$), and (**e**) real and (**f**) imaginary parts of $S_{22}^D$.

In order to examine the effect of $L_{nr}$ on the extracted $S_{11}^D$, $S_{21}^D$, $S_{12}^D$, and $S_{22}^D$, we also extracted these S-parameters for the constructed bianisotropic MM slab by our method using an NR-Line (an empty waveguide section) with $L_{nr} = 10.16$ mm. Figure 6a–f illustrate the extracted $S_{11}^D$, $S_{21}^D$ ($\cong S_{12}^D$), and $S_{22}^D$ after applying the RA procedure for frequency range of approximately 42 MHz. It is noted from Fig. 6a–f that the extracted $S_{11}^D$, $S_{21}^D$, and $S_{22}^D$ for $L_{nr} = 10.16$ mm are similar to those for $L_{nr} = 9.4$ mm given in Fig. 4a–f (with maximum variation less than 3%). This indicates not only the non-dependence of our method on $L_{nr}$ (provided that $P_0 \ne 1.0$) but also its stability.

## Extracted S-parameters of a sensing area

### The examined topology

**Figure 7:**
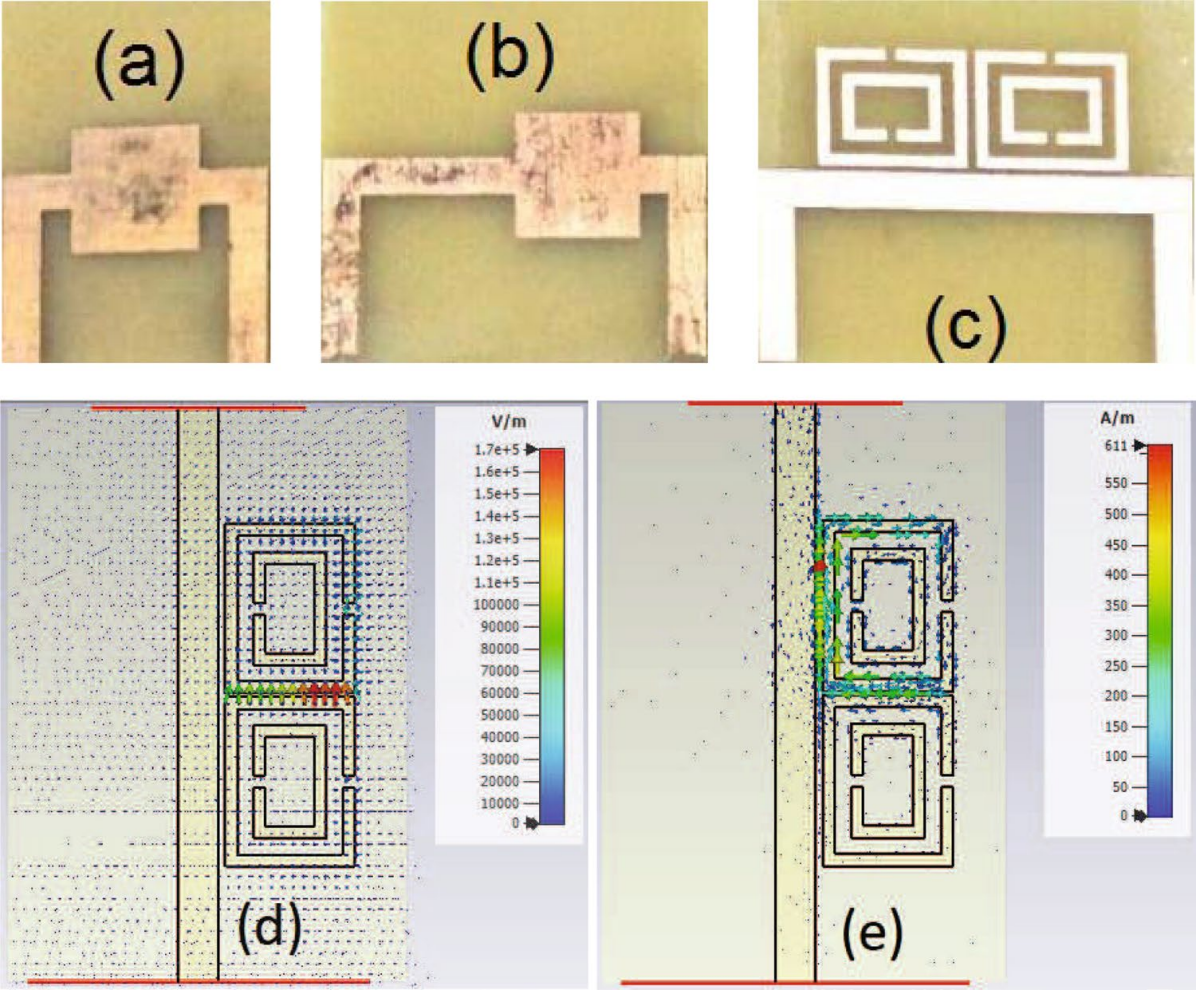
Fabricated microstrip lines: (**a**) The configuration of the R-Line in Fig. 2a, b the configuration of the NR-line next to the R-Line in Fig. 2b, c the configuration of the Device or the sensing area (double resonators next to the microstrip line) in Fig. 2c, and spatial distributions of (**d**) electric field (V/m) around the SRRs (side view) and (**e**) surface current (A/m) on the surface of the metals of SRRs (side view) at 2.193 GHz.

After validating our proposed method for a bianisotropic MM slab positioned into a waveguide section, we then proceeded with extraction of S-parameters of a sensing area (or a two-port network (line)) involving SRR resonators next to a microstrip line. Figure 7a–c illustrate photos of the fabricated configurations of a R-Line, a NR-Line, and a device with double SRR resonators next to the microstrip line (grounds are not shown for clarity).

The FR4 material with $\varepsilon _{r,sub} = 4.3 (1 - i 0.025)$ and thickness $d_{\text {sub}} = 1.6$ mm was used as a substrate material. Microstrip line, R-Line, NR-Line, Device, and ground were all constructed by the copper material ($\sigma = 5.8 \times 10^7$ S/m and $t_m = 35\mu$m). For the R-Line, microstrip line with a width of 10.0 mm and a length of $L_{r} = 9.7$ mm ($w_s = 3.0$ mm, see Fig. 1) was considered. This line having an effective relative dielectric constant of approximately $\varepsilon _{\text {eff}} \cong 3.618 - i 0.085$ and an effective impedance of approximately $Z_{\text {eff}} \cong 21.881 + i 0.258$ ohm^55^ introduces symmetric reflections on both sides of the microstrip line.

For the NR-Line, we considered a microstrip line with a width of $w_s = 3.0$ mm (see Fig. 1), and a length of $L_{nr} = 9.7$ mm. This line having an effective relative dielectric constant of approximately $\varepsilon _{\text {eff,0}} \cong 3.263 - i 0.074$ and an effective impedance of approximately $50.573 + i 0.571$ ohm^55^ produces essentially near-zero reflection. Figure 5b shows the magnitudes of simulated S-parameters of the configuration of the NR-line next to the R-Line in Fig. 7b. For microstrip measurements, the setup in Fig. 3c, except for the waveguide sections, was utilized. In the simulations, the Frequency-Domain solver of the CST Microwave Studio was utilized. Here, $E_t = 0$ was set at the ground, open boundary conditions with additional space was used on the top, and open boundary conditions (without additional space) were applied for all configurations in Fig. 7a–c. Waveguide ports whose dimensions were calculated using the built-in macro function of port extension coefficient were positioned at beginning of the microstrip lines. Adaptive mesh refinement was activated in the solver with an accuracy of $10^{-12}$ (3^rd^ order solver). It is seen from Fig. 5b that the configuration in Fig. 7b has reflection-asymmetric behavior ($|S_{11}| \ne |S_{22}|$).

For the device, two identical resonators (next to the microstrip line) cascaded in longitudinal direction were considered. The geometrical parameters of this device, as shown in Fig. 1, are as follows: $L_{r1} = 9.7$ mm, $L_{r2} = 12.7$ mm, $w = g = 0.9$ mm, $s = 1.2$ mm, $L_{g1} = 0.40$ mm, and $L_{g2} = 0.50$ mm. Besides, the geometrical parameters of the microstrip line section are $L_{s1} = 15.85$ mm, $L_{s2} = 12.85$ mm ($w_s = 3.0$ mm), and $L_{s3} = 2.15$ mm (the same for the R-Line and NR-Line configurations in Fig. 7a,b.

Besides, Fig. 7d,e illustrate, respectively, the spatial distributions of electric field around the SRRs (side view) and surface current on the surface of the metals of SRRs (side view) at 2.193 GHz where $|S_{21}|$ has a minimum value. It is seen from Fig. 7d,e that aside from circulating currents, which augment specially for the SRR segment near the main microstrip feedline) on the SRRs due to magnetic field effect (magnetic excitation), the proximity of the rings creates interaction (electric field coupling—Fig. 7d) and improves resonance characteristics of the double SRR configuration. Besides, Fig. 7d,e demonstrate the spatial distributions at the time that the upper SRR is mainly active. It should be pointed out that only one of the SRR is chiefy active while the other one behaves almost passive at critical time periods, thus sharing field interaction with time.

### Extracted S-parameters referenced to tapers/launchers

**Figure 8:**
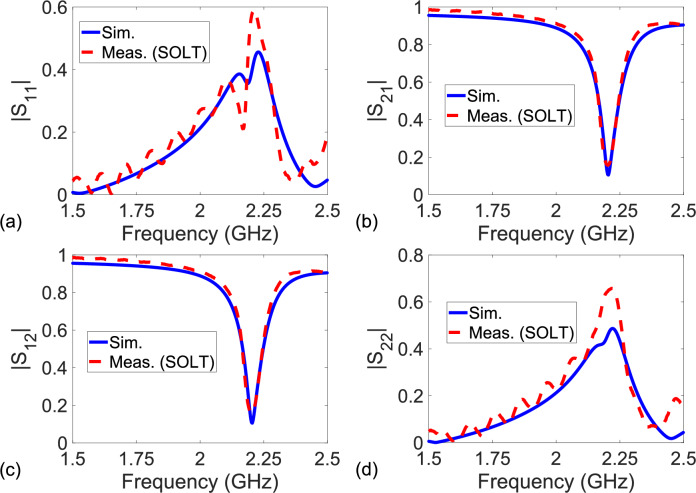
Magnitudes of simulated S-parameters (‘Sim.’ with solid lines) and measured S-parameters after the SOLT calibration technique (‘Meas. (SOLT)’ with dashed lines) referenced to tapers/launchers of the configuration in Fig. 1) over $1.5-2.5$ GHz: (**a**) $|S_{11}|$, (**b**) $|S_{21}|$, (**c**) $|S_{12}|$, and (**d**) $|S_{22}|$.

Before presenting extracted S-parameters ($S_{11}^D$, $S_{21}^D$, $S_{12}^D$, and $S_{22}^D$) of the sensing area in Fig. 1, it would be instructive to show S-parameters ($S_{11}$, $S_{21}$, $S_{12}$, and $S_{22}$) of the configuration in Fig. 1 referenced to tapers/launchers. Figure8a–d illustrate the magnitudes of simulated S-parameters (‘Sim.’ with solid lines) and measured S-parameters after the SOLT calibration (‘Meas. (SOLT)’ with dashed lines) over $1.5 - 2.5$ GHz. The measurement system was calibrated to tapers/launchers.

It is noted from Fig. 8a–d that $|S_{11}|$, $|S_{21}|$, $|S_{12}|$, and $|S_{22}|$ do not, respectively, correspond to $|S_{11}^D|$, $|S_{21}^D|$, $|S_{12}^D|$, and $|S_{22}^D|$ of the sensing area in Fig. 1 (double resonators next to the microstrip line). Furthermore, it is seen from Fig. 8a–d that although the simulated and measured $|S_{21}|$ and $|S_{12}|$ are in good agreement, the simulated and measured $|S_{11}|$ and $|S_{22}|$ have differences over the entire frequency band. There are three main mechanisms producing such a difference according to the configuration in Fig. 1. First, the SMA tapers or launchers used to transfer the coaxial line energy to the microstrip lines alter both magnitudes and phases of S-parameters. Second, microstrip feedline straights mainly influence phases of S-parameters. Third, microstrip feedline bends introduce changes chiefly in the magnitudes of S-parameters.

### Extracted S-parameters of the sensing area

**Figure 9:**
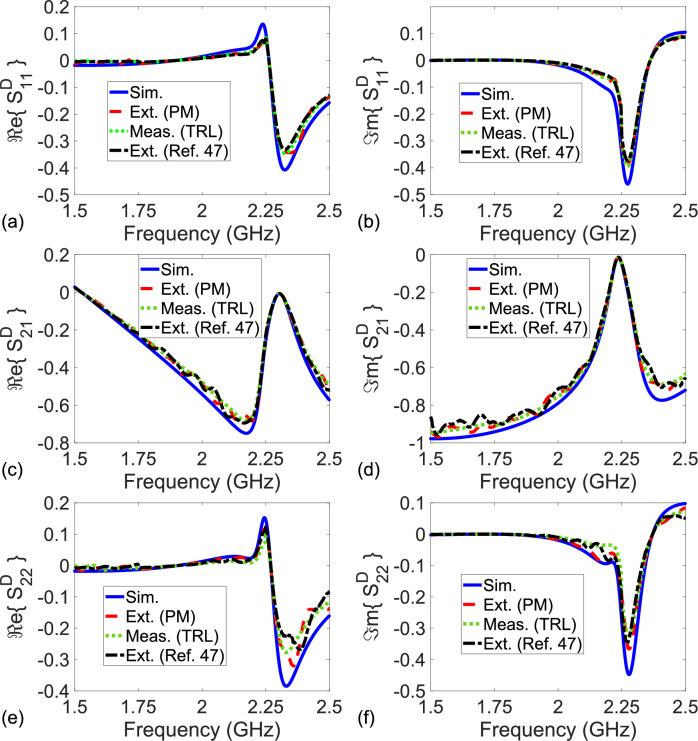
Simulated S-parameters (‘Sim.’ with solid lines), extracted S-parameters by the proposed method (‘Ext. (PM)’ with dashed lines), measured S-Parameters by the TRL calibration procedure^22^ (‘Meas. (TRL)’ with dotted green lines), and extracted S-parameters by the de-embedding method^47^ (‘Ext. (Ref. 47)’ with dashdot black lines) of the sensing area (double resonators next to the microstrip line). (**a**) Real and (**b**) imaginary parts of $S_{11}^D$, (**c**) real and (**d**) imaginary parts of $S_{21}^D$ ($\cong S_{12}^D$), and (**e**) real and (**f**) imaginary parts of $S_{22}^D$.

To eliminate the effect of the SMA connectors on measurements, and to extract only the S-parameters of the sensing area in Fig. 1, we implemented our proposed method using uncalibrated S-parameters of the configurations in Fig. 7a–c. Figure 9a–f show the extracted real and imaginary parts of $S_{11}^D$, $S_{21}^D$, $S_{12}^D$, and $S_{22}^D$ of the sensing area over 1.5-2.5 GHz (after applying the RA procedure to the extracted S-parameters for a frequency range of 10 MHz using $N_{\text {int}} = 10$ (selected as a higher value than the one used in the study^54^ to get more smoothed measurement data), $f_{\text {max}} = 2.5$ GHz, $f_{\text {min}} = 1.5$ GHz, and $N_f = 1001$^54^). Extracted S-parameters without the RA are not presented here for simplicity. For comparison, in addition to S-parameter simulations, we applied the TRL calibration procedure^22^ and the de-embedding methods^46–49^. It is noted that the de-embedding method^46^ is restricted to $S_{21}^{D}$ and $S_{12}^D$ only. In implementation of the TRL calibration procedure, a calibration kit designed using an FR4 substrate ($\varepsilon _{r,sub} = 4.3 (1 - i 0.025)$ and $d_{\text {sub}} = 1.6$ mm), as shown in Fig. 3d, was utilized. For the thru standard, a 60 mm microstrip line ($w_s = 3.0$ mm and $Z_{\text {eff}} \cong 50$ $\Omega$) was used. For the line standard, a 73.83 mm microstrip line ($w_s = 3.0$ mm and $Z_{\text {eff}} \cong 50$ $\Omega$), which corresponds to an effective length of 13.83 mm in reference to the thru standard. This line standard, in reference to the thru standard, will produce an effective bandwidth of 4.44 GHz (between 560 MHz and 5.0 GHz), within which the line phase undergoes a maximum change of ${\mp } 90^o$^56^. The reflect line was implemented by a well-soldered via. An additional microstrip with a sufficient length of 30 mm was used for all standards to measure smoother S-parameters after the TRL calibration.

Among the applied methods^22^ and^46–49^, only the results from the methods in^22^ and^47^ are presented in Fig. 9a–f for a clear view. A quantitative analysis of all the methods^22^ and^46–49^ will be presented shortly. It is noted from Fig. 9a–f that the simulated, measured, and extracted S-parameters of the sensing area are close each other over the entire band. We think that small oscillations observed in the extracted real and imaginary parts of $S_{11}^D$, $S_{21}^D$ ($\cong S_{12}^D$), and $S_{22}^D$ of the sensing area might be partly due to tolerances in the fabricated configurations of the R-Line, the NR-Line, and the sensing area. For a quantitative analysis for how well the extracted or measured S-parameters approach the simulated ones, we calculated the root-mean-square error (RMSE) values for all considered methods using

41 $$\begin{aligned} \chi _{\text {RMSE}} = \sqrt{ \frac{1}{N_f} \Bigg [ \sum _{k = 1}^{N_f} \left( \chi _k^{\text {ref}} - \chi _k^{\text {ext/meas}} \right) ^2 \Bigg ] }, \end{aligned}$$

where $\chi$ stands for $\Re e\{ S_{11}^D \}$, $\Re e\{ S_{21}^D \}$, $\Re e\{ S_{12}^D \}$, $\Re e\{ S_{22}^D \}$, $\Im m\{ S_{11}^D \}$, $\Im m\{ S_{21}^D \}$, $\Im m\{ S_{12}^D \}$, or $\Im m\{ S_{22}^D \}$; and $\chi _k^{\text {ref}}$ and $\chi _k^{\text {ext/meas}}$ are the reference (simulated) and extracted/measured $\chi$ values at the *k*th frequency.

Table 2 presents the calculated RMSE values of the measured or extracted $\Re e\{ S_{11}^D \}$, $\Re e\{ S_{21}^D \}$, $\Re e\{ S_{12}^D \}$, $\Re e\{ S_{22}^D \}$, $\Im m\{ S_{11}^D \}$, $\Im m\{ S_{21}^D \}$, $\Im m\{ S_{12}^D \}$, and $\Im m\{ S_{22}^D \}$. It is seen from Table 2 that the extracted S-parameters of our method and the measured ones of the TRL calibration procedure are similar, and both are much closer to the simulated S-parameters than the extracted S-parameters of the methods in^46–49^. For instance, while RMSE values of $\Re e\{S_{11}^D\}$ and $\Im m\{S_{11}^D\}$ are, respectively, around 0.0271 and 0.0279 for our method, those of the de-embedding technique^48^ approach as high as 0.0318 and 0.0324. Besides, the accuracy of our method depends on whether $P_0$ approaches unity, as discussed in Section *The Analysis of the Method*. Figure 5a demonstrates the dependence of the real and imaginary parts of $P_0$ of the NR-Line with $L_{nr} = 9.7$ mm over $1.5 - 2.5$ GHz. It is seen from Fig. 5a that $P_0$ does not approach unity over the entire band.

**Table 2:** Calculated RMSE values of $\Re e \{ S_{11}^D \}$, $\Re e \{ S_{21}^D \}$, $\Re e \{ S_{12}^D \}$, $\Re e \{S_{22}^D \}$, $\Im m\{ S_{11}^D \}$, $\Im m\{ S_{21}^D \}$, $\Im m\{ S_{12}^D \}$, and $\Im m\{ S_{22}^D \}$ for the methods^22,46–49^ and our proposed method.

RMSE	Calibration or De-embedding Technique					
values	^22^	^46^	^47^	^48^	^49^	PM
$\Re e \{ S_{11}^D \}$	0.0260	–	0.0289	0.0318	0.0281	0.0271
$\Re e \{ S_{21}^D \}$	0.0199	0.0215	0.0218	0.0241	0.0208	0.0206
$\Re e \{ S_{12}^D \}$	0.0196	0.0219	0.0219	0.0241	0.0207	0.0202
$\Re e \{ S_{22}^D \}$	0.0254	–	0.0291	0.0306	0.0277	0.0264
$\Im m \{ S_{11}^D \}$	0.0265	–	0.0295	0.0324	0.0289	0.0279
$\Im m \{ S_{21}^D \}$	0.0201	0.0212	0.0222	0.0217	0.0211	0.0203
$\Im m \{ S_{12}^D \}$	0.0199	0.0218	0.0217	0.0244	0.0209	0.0200
$\Im m \{ S_{22}^D \}$	0.0262	–	0.0282	0.0315	0.0284	0.0271

### Advantages and disadvantages of the proposed method

Table 1 presents a comparison of our method with two calibration techniques (SOLT and TRL (or LRL))^21,22^ and with other de-embedding techniques in the studies^46–49^ in terms of the need for error network analysis, the total number of standards used in their implementation, capability of full two-port characterization, the possibility of any sign ambiguity, realization of standards, and requirement of a new design if a new two-port network or line is utilized. The following points are noted from the results in Table 1. First, our de-embedding technique, just as other de-embedding techniques in the studies^46–49^, does not require determination of error networks in the characterization of a two-port network (transmission line or sample), whereas calibration techniques SOLT and TRL (or LRL) (as well as other calibration techniques) do require this determination. Second, while our method and the de-embedding techniques in the studies^46–49^ necessitate two different standards in their implementation, the calibration techniques SOLT and TRL (or LRL) (as well as other calibration techniques) need at least three different calibration standards for their application. Third, our de-embedding technique, the de-embedding techniques in the studies^47–49^, and calibration techniques SOLT and TRL (or LRL) can perform full two-port characterization. Nonetheless, the de-embedding technique^46^ is limited to $S_{21}$ and $S_{12}$ only. Fourth, our de-embedding technique together with calibration techniques SOLT and TRL (or LRL) do not have any sign ambiguity in the full characterization procedure (determining all S-parameters) of a two-port network or line. On the other hand, the de-embedding techniques^46–49^ could have such an ambiguity problem. Fifth, while standards of our de-embedding technique and other de-embedding techniques along with the calibration technique^22^ are relatively easier to realize than those of the calibration technique^21^, because the realization of the open standard could be partly harder. It should be pointed out here that as the calibration techniques SOLT and TRL (or LRL) (as well as other calibration techniques), the accuracy of our proposed method and the de-embedding techniques^46–49^ is mainly related to non-unity value of $P_0$. To eliminate this disadvantage, as a rule of thumb, shorter NR-Lines, which can be arranged in the design procedure once the frequency range is specified, should be used to remove this possibility. Finally, the proposed method and the de-embedding techniques^46–49^ share the common problem of the requirement of a new design if the two-port line modifies (e.g., if the feedline of the microstrip line changes). The calibration techniques SOLT and TRL (or LRL) (as well as other calibration techniques) do not have such a problem. Nonetheless, such a drawback is not the main issue in the sensing area characterization of sensors since, once designed, optimized, and then fabricated, these sensors are utilized only for a precise application^13–20^.

## Conclusion

A method is proposed to determine the S-parameters of two-port devices (or networks) using uncalibrated S-parameter measurements at microwave frequencies. The method requires the use of non-reflecting line and reflecting line standards (partially unknown) and determines uniquely all S-parameters of a two-port device without the need for evaluating error coefficients or networks. The method is first validated by S-parameters of a bianisotropic MM slab (constructed by square-shaped SRRs embedded into a waveguide) as the first device. After, it is tested for extracting $S_{11}^D$, $S_{12}^D$, $S_{21}^D$, and $S_{22}^D$ of a sensing area involving double SRRs next to a microstrip line. The TRL calibration procedure and four different de-embedding techniques, supported by S-parameter simulations, were applied to examine the accuracy and performance of our method. Our method, however, requires measurements of two (direct and reversed) configurations of the device. Eliminating this need will be considered for a future study.

## Data Availability

The datasets used and/or analysed during the current study are available from the corresponding author (U.C.H.) on reasonable request.
